# EasyEyes — A new method for accurate fixation in online vision testing

**DOI:** 10.3389/fnhum.2023.1255465

**Published:** 2023-11-29

**Authors:** Jan W. Kurzawski, Maria Pombo, Augustin Burchell, Nina M. Hanning, Simon Liao, Najib J. Majaj, Denis G. Pelli

**Affiliations:** ^1^Department of Psychology, New York University, New York, NY, United States; ^2^Institut für Psychologie, Humboldt Universität zu Berlin, Berlin, Germany; ^3^Center for Neural Science, New York University, New York, NY, United States

**Keywords:** fixation, online testing, eye tracker, crowding, EasyEyes, crosshair tracking, gaze control

## Abstract

Online methods allow testing of larger, more diverse populations, with much less effort than in-lab testing. However, many psychophysical measurements, including visual crowding, require accurate eye fixation, which is classically achieved by testing only experienced observers who have learned to fixate reliably, or by using a gaze tracker to restrict testing to moments when fixation is accurate. Alas, both approaches are impractical online as online observers tend to be inexperienced, and online gaze tracking, using the built-in webcam, has a low precision (±4 deg). EasyEyes open-source software reliably measures peripheral thresholds online with accurate fixation achieved in a novel way, without gaze tracking. It tells observers to use the cursor to track a moving crosshair. At a random time during successful tracking, a brief target is presented in the periphery. The observer responds by identifying the target. To evaluate EasyEyes fixation accuracy and thresholds, we tested 12 naive observers in three ways in a counterbalanced order: first, in the laboratory, using gaze-contingent stimulus presentation; second, in the laboratory, using EasyEyes while independently monitoring gaze using EyeLink 1000; third, online at home, using EasyEyes. We find that crowding thresholds are consistent and individual differences are conserved. The small root mean square (RMS) fixation error (0.6 deg) during target presentation eliminates the need for gaze tracking. Thus, this method enables fixation-dependent measurements online, for easy testing of larger and more diverse populations.

## Introduction

Online data collection offers researchers immediate access to thousands of participants around the world, which speeds up research and allows for more diverse samples (Palan and Schitter, 2018; Grootswagers, 2020). However, for researchers conducting visual fixation-dependent experiments, the appeal of online testing is frustrated by the inability to track gaze precisely. This is especially important when stimuli are presented in the periphery.

In peripheral testing, observers are torn between fixating on the central crosshair and looking toward the anticipated target location, which we call “peeking” (Kurzawski et al., 2023). If observers fixate on the anticipated location of the peripheral target, target eccentricity is almost zero, defeating the purpose of peripheral testing. In-lab eye tracking is widely used to ensure fixation. Typically, infrared light emitted by the eye tracker creates a reflection in the cornea of the eye, which is picked up by an infrared camera (Eye Tracking 101, 2022). Thus, eye trackers can precisely report the eye’s location and movement at any point in time. However, precise eye trackers are generally expensive, cumbersome, and require calibration. Importantly, they limit the study of fixation-dependent experiments to laboratory settings.

Precise gaze control is not available for online testing yet. Even though many researchers have devised tools and methods to gather eye-tracking data using participants’ webcams (Xu et al., 2015; Huang et al., 2016; Valliappan et al., 2020), many of these tools still require calibration. An exception is *WebGazer.js* (Papoutsaki et al., 2016), a prominent auto-calibrated eye-tracking tool that relies on the webcam to estimate the participant’s gaze. Researchers have shown its effectiveness for various tasks (Semmelmann and Weigelt, 2018; Slim and Hartsuiker, 2022). Nevertheless, in the best-case scenario, its spatial accuracy is approximately 4 deg, which would introduce a ± 40% error in the eccentricity of a target at 10 deg eccentricity (Papoutsaki, 2015; Huang et al., 2016).

Ample research on eccentricity-based and polar angle-based differences in perception (see Strasburger et al., 2011; Himmelberg et al., 2023 for reviews) relies on stable central eye fixation (Guzman-Martinez et al., 2009). Visual crowding experiments are well-known fixation-dependent psychophysical tasks. Crowding, or the failure to recognize an object due to clutter, is typically measured by asking participants to recognize a letter between two flankers (Stuart and Burian, 1962; Bouma, 1973; Pelli et al., 2004; Pelli and Tillman, 2008; Strasburger, 2020). *Crowding distance* (“critical spacing”) is the center-to-center distance from target to flanker that achieves a criterion level of performance. It increases with eccentricity, and thus, crowding is generally measured in the periphery (Bouma, 1970; Toet and Levi, 1992; Kooi et al., 1994; Pelli et al., 2004; Levi and Carney, 2009).

Crowding varies 2-fold across observers (Pelli et al., 2016; Kurzawski et al., 2023) and little within an observer for a given eccentricity and polar angle across sessions (Chung, 2007; Kurzawski et al., 2023). Clinically, it plays a key role in amblyopia (Levi et al., 2007) and exacerbates the effects of macular degeneration (Wallace et al., 2017). It correlates with dyslexia and thus may be a valuable biomarker to guide early interventions designed to diminish problems in decoding letters and words (Levi, 2008; Joo et al., 2018; Li et al., 2020). For crowding to fulfill its promise as a biomarker, accurate target eccentricity when testing is required. We have previously shown that measured crowding distance depends on fixation accuracy and inaccurate fixation impacts the mean and standard deviation of measured crowding thresholds (Kurzawski et al., 2023). One way to avoid inaccurate fixation is gaze-contingent stimulus presentation that here we call “awaited fixation”: While monitoring gaze with an eye tracker, the stimulus only appears after the observer has accurately fixated for 250 ms. Unfortunately, online gaze tracking is not accurate enough to use this method.

Here, we demonstrate how EasyEyes,^1^ an open-source online psychophysical testing tool, measures crowding thresholds reliably by achieving accurate fixation with a fine motor task and without eye tracking.

Researchers have shown that cursor movement generally correlates with eye movement (Chen et al., 2001; Liebling and Dumais, 2014). Moreover, looking at a target is required for precise and accurate hand movements (Jana et al., 2017), and fixations are necessary when coordinating the movement between two objects (Land and Hayhoe, 2001). With EasyEyes, observers perform the fine motor task of tracking a moving crosshair with their cursor. The peripheral target is presented after successful crosshair tracking for a random time of 0.75–1.25 s. This eye–hand coordination task demands accurate fixation before target onset.

We compare thresholds between an at-home online crowding task (EasyEyes home), an in-lab version of the same online task (EasyEyes lab), and a previously validated crowding in-lab task (CriticalSpacing.m lab, Pelli et al., 2016). We find that online EasyEyes crowding thresholds do not significantly differ from those measured in the laboratory. Additionally, we use gaze tracking while observers complete EasyEyes in the lab to validate that observers fixate on the moving crosshair during target presentation and do not peek.

## Methods

### Observers

Twelve observers took part in our experiment. Seven identified as female and five as male. Their ages ranged from 21 to 46 (*M* = 27.3, *SD* = 6.8). All observers were fluent English speakers and had normal or corrected-to-normal vision. Importantly, observers were recruited via a convenience sample, ensuring that they had little to no experience with crowding tasks. Two-thirds of the observers were associated with the psychology department of New York University (graduate students, postdocs, and staff), but had no experience with vision psychophysical tasks. All observers gave informed consent in accordance with the Declaration of Helsinki and were compensated $15/h for their participation. This experiment was approved by the New York University Committee on Activities Involving Human Subjects (UCAIHS; IRB-FY2016-404).

All observers completed a visual crowding task in three ways: 1. CriticalSpacing.m (in lab), 2. EasyEyes (in lab), and 3. EasyEyes (at home). The order of the three ways was counterbalanced across observers to cancel out any order effects.

### Way 1: CriticalSpacing.m in lab

CriticalSpacing.m (Pelli et al., 2016) is a thoroughly tested MATLAB program for measuring crowding thresholds, recently enhanced by the addition of a chin rest and gaze-contingent display (Kurzawski et al., 2023). The target (with flankers) is presented when gaze is detected within 1.5 deg of the crosshair by an EyeLink 1000 eye tracker. Trials are retained only if fixation remains within 1.5 deg of the crosshair throughout the target duration. Using this method, Kurzawski et al. (2023) report extensive crowding measurements on 50 observers.

### Way 2: Easyeyes in lab

Observers used the same chin rest, and EasyEyes online software measured the crowding threshold while the EyeLink 1000 independently monitored gaze. EasyEyes software had no access to the gaze data. An EasyEyes log recorded a time stamp in absolute POSIX time (in fractions of a second), and the crosshair, cursor, and target position every frame (60 Hz). A MATLAB program running in parallel saved a POSIX timestamp and gaze position every 10 ms.

### Way 3: Easyeyes at home

Each observer opened the URL of the EasyEyes experiment in a browser on their own computer and ran the experiment online.

Observers who initially completed CriticalSpacing.m in the laboratory or EasyEyes in the laboratory may have inferred that the task required strict fixation, potentially biasing their subsequent fixation performance on EasyEyes at home. We therefore counterbalanced the order in which observers completed the conditions.

### Identification task

In all testing methods, observers completed a simple letter recognition task that measures crowding in the visual periphery. In each trial, the observer is presented with a trigram of letters for 150 ms. We refer to the middle letter as the target and the other two as the flankers. For each trial, target and flankers are drawn randomly, without replacement, from a nine-letter character set: DHKNORSVZ. Letters are rendered in black in the Sloan font on a uniform white background of approximately 275 cd/m^2^ (Sloan et al., 1952; Pelli et al., 2016). We omit the C from Louise Sloan’s original 10 letters because it is too easily confused with the O (Elliott et al., 1990). The Sloan letters all have the same square (invisible) bounding box. The target letter is presented so that its center is either −10 deg or + 10 deg from the fixation crosshair along the horizontal midline. The flankers are presented symmetrically, to the right and left of the target. The spacing, center of target to center of each flanker varies from trial to trial, guided by QUEST. After the brief presentation, the list of nine possible letters is displayed, and the observer is asked to identify the target by clicking (or typing, in the case of the EasyEyes home session) one of the nine letters displayed. Only the valid characters (nine Sloan letters) are accepted as responses, and any other keypress is ignored.

As our observers were naive to the task, they completed a brief (2–3 min) online training session which consisted of 10 trials, 5 at each of ±10 deg of eccentricity, prior to any session.

### Measuring threshold

In each block, we use QUEST (Watson and Pelli, 1983) to control the letter spacing of each trial and finally estimate the crowding distance threshold. Each threshold estimate was based on 35 trials. Each block of trials interleaved two conditions, one for −10 deg and another for +10 deg (resulting in 35 trials per condition and 70 trials per block). Each participant completed two blocks in each session (140 trials per session). Letter size scales with spacing, maintaining a fixed ratio of 1.4:1 (in EasyEyes, *spacingOverSizeRatio* = 1.4). Threshold was defined as the letter spacing for 70% correct identification, and QUEST assumes a Weibull function. In EasyEyes, we specify *thresholdProportionCorrect* as 70, *thresholdParameter* as “spacing,” and *thresholdGuess* to be 3. The remainder threshold parameters conserved their default values (*thresholdDelta* is 0.01, *thresholdBeta* is 2.3, and *thresholdGamma* is 0.5). These values match those of CriticalSpacing.m (Kurzawski et al., 2023).

The conditions for target presentation differ depending on the experimental software. CriticalSpacing.m uses gaze-contingent stimulus presentation while EasyEyes relies on crosshair tracking. These are described below.

### Gaze-contingent CriticalSpacing.m

We measured crowding thresholds using CriticalSpacing.m (Pelli et al., 2016) with additional features that integrated compatibility with the EyeLink eye tracker. This enhanced CriticalSpacing.m uses gaze-contingent stimulus presentation that we call “awaited fixation” (Kurzawski et al., 2023). At the start of the experiment, a central crosshair is shown on the screen. The first trial begins when the observer presses the spacebar. After correct fixation for 250 ms, a letter trigram is displayed for 150 ms. After the stimulus offset, the observer uses a mouse to click to report the middle letter of the trigram. All possible letters appear in a row below fixation. After clicking on the letter, the observers are instructed to look back at the central crosshair. A correct response is acknowledged with a short beep. Subsequently, the computer waits for the observer to maintain fixation within 1.5 deg of the crosshair for 250 ms. If the waiting period exceeds 10 s, software prompts for recalibration of the gaze tracker.

### Apparatus

In the laboratory, observers used a chin rest to maintain a 40 cm viewing distance from eye to display. To track gaze in the laboratory, we used an EyeLink 1000 eye tracker (SR Research, Ottawa, Ontario, Canada) with a sampling rate of 1,000 Hz. To allow for a short viewing distance, we used their Tower mount setup with a 25-mm lens.

Each in-lab session was completed with an Apple iMac 27″ with an external monitor for stimulus presentation. The screen resolution was 5,120 × 2,880. Apple iMac has AMD graphics for optimal compatibility with Psychtoolbox imaging software. The Systems Preference: Displays: Brightness slider was set (by calling MacDisplaySettings.m in the Psychtoolbox) to 0.86 (range 0 to 1) to achieve a white background luminance of approximately 275 cd/m^2^. The observer viewed the screen binocularly. Stimuli were rendered using *CriticalSpacing*.*m* software (Pelli et al., 2016) implemented in MATLAB 2021 using the Psychtoolbox (Brainard, 1997).

### EasyEyes

EasyEyes (see text footnote 1) is open-access software to measure thresholds online. With a Pavlovia^2^ account, the scientist can upload an experiment table with an alphabetical list of parameters along with corresponding files (consent forms and fonts) to the EasyEyes website and obtain an experiment link. EasyEyes integrates Prolific^3^ to allow scientists to easily recruit paid participants from all over the world. After participants complete the experiment, EasyEyes provides easy access to the data as well as tools for data analysis and visualization.

EasyEyes has 305 parameters that allow scientists flexibility to include questionnaires and measure various variables, including reading speed and accuracy, visual acuity, and hearing audiogram. EasyEyes uses the “virtual chinrest” method of Li et al. (2020) to measure screen size and viewing distance and uses Google FaceMesh (Kartynnik et al., 2019) to continuously track viewing distance throughout the experiment.

### Experimental design

For the EasyEyes version of the letter identification task, we implement the CriticalSpacing.m task described above as closely as possible. Our spreadsheet specifies 3 blocks of two target tasks: one *questionAndAnswer* block (that asks observers for their participant ID and age) and two *identify* blocks. Each *identify* block has two interleaved conditions of 35 trials each. The only difference between the conditions is whether the target position is specified at ±10 deg (*targetEccentricityXDeg* = 10 or − 10 and *targetEccentricityYDeg* = 0). In this way, each block calculates two thresholds, one for the right and one for the left meridian. We specify the threshold criterion proportion correct (70%), the viewing distance (40 cm), and the stimulus presentation time (0.15 s) using the *thresholdProportionCorrect*, *viewingDistanceDesiredCm*, and *targetDurationSec* parameters, respectively. We specify *targetKind* to be “letter,” *spacingDirection* to be “radial,” *spacingRelationToSize* to be “ratio,” and *spacingSymmetry* to be “screen.” We also provide software with the WOFF2 file of the Sloan font and indicate it as such using the *font*, *fontSource*, and *fontCharacterSet* parameters.

There are three differences between the at-home and in-lab EasyEyes experiments. First, the in-lab version sets the *_trackGazeExternallyBool* parameter to TRUE to save a log of timestamped screen locations of the crosshair, cursor, and (when present) target. Second, the at-home experiment requires observers to calibrate their screen size and viewing distance as described above. Finally, in the at-home experiment, the *viewingDistanceNudgingBool* parameter is set to TRUE so observers are told to move farther away from or closer to the screen if their viewing distance is less than 80% or greater than 120% of the specified 40 cm.

### Moving crosshair

Traditionally, many vision experiments ask observers to fix their gaze on a static fixation mark, which is often a crosshair. Naive observers struggle to hold their gaze on a central mark while anticipating a peripheral target. Instead, EasyEyes tells the observer to use the cursor to track a moving crosshair.

Each trial presents a moving black crosshair consisting of a vertical and a horizontal line crossing at their midpoints, each 2 deg long and 0.05 deg thick. The crosshair has an invisible “hotspot” disk with a 0.1 deg radius about its center location. Until stimulus presentation, the crosshair moves steadily, counterclockwise, along an invisible circular trajectory centered on the screen center, with a radius of 0.5 deg and a period of 10 s, resulting in a speed of 0.3 deg/s. The initial position of the crosshair is a random point on the circular path. The observer is told to use the cursor to track the center of the crosshair. Tracking is considered successful while the cursor tip is in the hotspot, and the crosshair becomes “bold” (increasing line thickness from 0.05 to 0.07 deg) while tracking is successful. This feedback helps the participant to quickly learn tracking.

In each trial, the tracking period is a small fraction of the circle. If the observer is tracking continuously, the average tracking duration is 1 s, which corresponds to 9% of the circle. The arc angle of the path is so small that the path curvature is hardly noticeable. In a limited exploration of hotspot radius, crosshair speed, and radius of curvature of crosshair movement, we settled on a hotspot radius of 0.1 deg (which yields excellent overall performance, RMSE <0.1 deg), a crosshair speed of 0.3 deg/s, and a radius of 0.5 deg. We explored different values but found that observers became frustrated by smaller radii or higher speeds. Similarly, an unpredictable “random walk” might produce more precise fixation, but would similarly frustrate observers or steal attention from the target. More investigation of these parameters is warranted.

### Coordinates

This study uses two spatial coordinate systems to specify stimulus and gaze position: *Screen coordinates* (pix) are X and Y pixels, with the origin in the upper left corner of the screen (and y increases down). *Visual coordinates* (deg) are X and Y gaze positions relative to the current location of the crosshair (and y increases up). The target is presented at 10 deg left or right of the crosshair center. (Stimulus display software needs to convert back and forth between these coordinate systems. The Supplementary material provides these routines in MATLAB and JavaScript.)

### Pre-stimulus interval

Before the first trial, participants received verbal instruction that they should use a mouse or trackpad to track the moving cross and should identify the middle letter presented in the periphery. At the start of each trial, EasyEyes displays the instruction in the upper left corner of the screen, “Ready? Track the center of the crosshair until the letter(s) appear.” (In order to avoid ambiguity in what is meant by “tracking,” we have since changed the instructions to “Ready? Use the cursor to track the center of the crosshair until the target appears. The crosshair becomes bold when the cursor is on it.”) We hypothesized that keeping the cursor near the crosshair forces the observer to fixate near the crosshair. Meanwhile, EasyEyes displays the cursor and moving crosshair, and checks the cursor position every frame until the cursor tip is in the crosshair hotspot; then, it starts a timer, which waits for a duration randomly sampled from a uniform probability interval of 0.75 to 1.25 s. EasyEyes checks the cursor position again at the end of the (unannounced) tracking interval. If the cursor tip is in the hotspot then EasyEyes presents the target. Otherwise, EasyEyes restarts the timer, without disturbing the crosshair’s motion. (Since collecting the data presented here, we made the cursor-tracking criterion more stringent. Now the cursor is checked repeatedly during the tracking interval and any exit from the hot spot causes EasyEyes to go back to the start.)

### Stimulus and response

The target (with flankers) is displayed immediately, for 150 ms. During the target presentation, the crosshair and cursor are hidden. 700 ms after the stimulus offset (specified by *targetSafetyMarginSec*), the following instructions appear: “Please identify the middle letter by clicking it below or pressing it on the keyboard.”

### Monitoring gaze while testing with EasyEyes

EasyEyes does not have gaze tracking. We use an in-lab EyeLink 1000 eye tracker to assess the accuracy of fixation during crosshair tracking by the cursor. A simple handshake between EasyEyes and MATLAB (controlling the EyeLink 1000) tells MATLAB when the experiment begins and ends. The handshake uses a RESTful API to a Node.js server. EasyEyes sends a start command by making a POST request to the RESTful server. Meanwhile, MATLAB repeatedly sends GET requests to the server until it receives the “start” command from EasyEyes. For each display frame (60 Hz), EasyEyes records the POSIX (absolute time in fractions of a second) timestamp, and the X and Y screen positions of the crosshair, cursor, and target. MATLAB receives X and Y gaze position from the EyeLink every 10 ms and records it along with a POSIX timestamp. This produces two timestamped CSV files, one from MATLAB and one from EasyEyes, which were combined to generate the plots seen here.

### RMSE of gaze and cursor position

We estimated RMSE by calculating the radial distance between either cursor and crosshair positions (tracking error) or gaze and crosshair positions (gaze pursuit error) for each frame of the stimulus presentation. These errors were averaged within and across trials to produce RMS errors per observer. In both cases (tracking and gaze pursuit errors), we report the mean error across observers.

### Correction of eye tracker calibration offset in X-Y gaze position

The eye tracker is calibrated once before the session by asking the observer to fixate in the center and at 5 deg above, below, right, and left from the center. We find that the center of the screen is reported with a small consistent offset unique to each observer session. We estimated and removed this offset. A correction was determined independently for each observer session by calculating the mean X and Y offset between crosshair and recorded gaze position across all gaze samples obtained during the 750 ms interval before stimulus onset (75 gaze samples per trial, and 140 trials). This single offset correction was applied to every gaze position of the observer in that session. Across observers, the mean ± SD RMS radial offset was 0.64 ± 0.25 deg.

### Statistical analysis of crowding thresholds

Test–retest correlation was assessed between log crowding distances. We also calculate the test–retest variance (which is reported as SD) as the square root of mean variance across observers. To evaluate the difference between methods, we conducted a one-way ANOVA with log crowding distance as the dependent variable and method (CriticalSpacing.m lab, EasyEyes lab, and EasyEyes home) as the independent variable and calculated a corresponding Bayes Factor using the anovaBF() function of the BayesFactor package in R (Morey et al., 2023). Furthermore, we evaluate the difference in log crowding threshold variance with pairwise F-tests for equal variance. To assess whether individual differences are conserved across methods, we compute each observer’s geometric mean threshold (4 thresholds: left and right, test and retest) and calculate the Pearson’s correlation coefficient for all pairs of methods. To assess how well observer differences are conserved we computed Pearson’s correlations (of the geometric mean across left and right meridians) across methods and across test and retest within each method. For example, to compare CriticalSpacing.m lab to EasyEyes home, we correlate the test thresholds in the former to the retest thresholds in the latter, and vice versa. We also calculate the intraclass correlation coefficient across all three methods. All analyses were conducted in R (version 4.2.3) using R Studio.

## Results

### Crowding thresholds agree across test–retest

**Figure 1:**
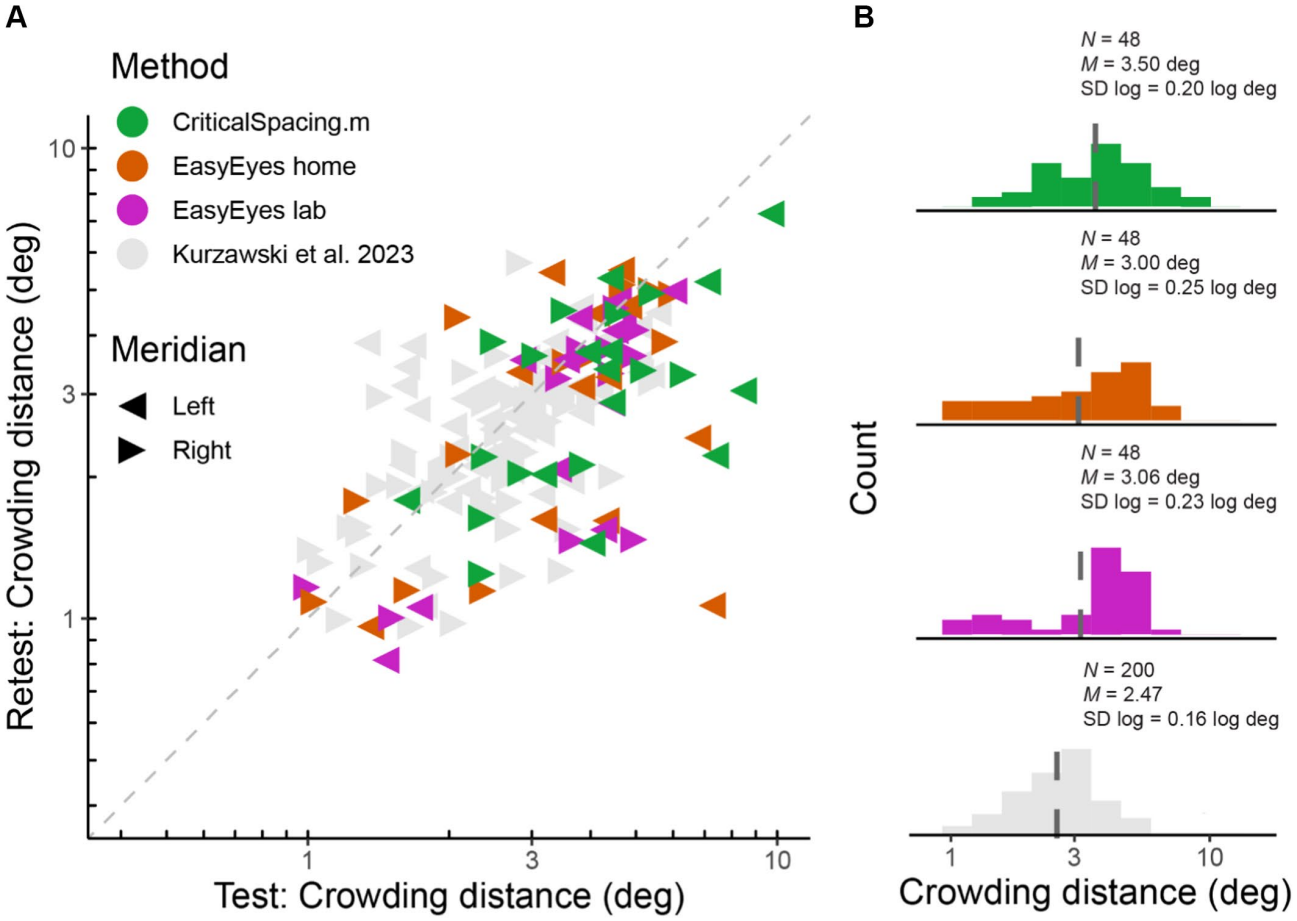
Log crowding thresholds across methods. **(A)** Test–retest crowding distances across methods. Gray triangles are thresholds measured by Kurzawski et al. (2023), and colored triangles are newly acquired data. Axes are log–log. **(B)** Histograms of log thresholds across methods. *M* is a geometric mean (dashed line) and SD is the standard deviation of all measured log crowding distances. *N* is the number of observations (12 observers, two meridians, test and retest) for our data and for fraction of data from Kurzawski et al. (50 observers, two meridians, test–retest).

We measured radial crowding thresholds in 12 observers on the right and left meridian at 10 deg of eccentricity using three experimental methods (EasyEyes home, EasyEasy lab, and CriticalSpacing.m lab). To assess the reliability of each method, we tested each threshold twice in two blocks separated by a break. We find that for all methods the test–retest correlations are highly significant (*p* < 0.01). The test–retest standard deviation was similar across methods (Figure 1A) and was not different from the results for 50 observers tested by Kurzawski et al. (2023) in an in-lab setting. Summary of standard deviations for our three methods and Kurzawski et al. (2023) are shown in Table 1. Figure 1A directly compares test and retest thresholds across methods. In each method, the retest over test ratio of crowding distance was approximately 0.8, slightly smaller than 0.9 in previous reports (Chung, 2007; Kurzawski et al., 2023). The improvement of the second session was independent of which method was used first to test the observer. Overall, crowding thresholds based on one 35-trial QUEST staircase have similarly good reproducibility across all three methods.

**Table 1:** Comparing test–retest thresholds for all methods.

Method of measuring crowding distance test–retest	Test–retest SD	Pearson’s *r*	Pearson’s *p*	Ratio
CriticalSpacing.m lab	0.16	0.58	<0.01	0.78
EasyEyes (home)	0.18	0.53	<0.01	0.82
EasyEyes (lab)	0.14	0.76	<0.01	0.79
Kurzawski et al. (2023)	0.11	0.55	<0.01	0.94

Test–retest SD represents the mean across observers of the standard deviation of test and retest of log crowding distance.

### Crowding thresholds agree across methods

Crowding varies across observers and very little across sessions within an observer (Chung, 2007; Kurzawski et al., 2023). While it is important to assess crowding’s reproducibility within a testing session or experiment, the core of this study is to compare crowding thresholds across three methods. Despite the variations between these methods, we find no significant differences between their measured thresholds. A one-way ANOVA shows no significant difference in mean log crowding threshold estimates, *F*(2) = 1.19, *p* = 0.308 The corresponding Bayes factor (BF01 = 5.4) indicates substantial evidence for the null hypothesis, which states no difference between the testing methods. Pairwise F-tests of equal variance show no significant difference in the log variance across methods: *F*(47, 47) = 1.26, *p* = 0.429 (EasyEyes lab vs. CriticalSpacing.m lab), *F*(47, 47) = 1.49, *p* = 0.173 (EasyEyes home vs. CriticalSpacing.m lab), and *F*(47, 47) = 0.85, *p* = 0.566 (EasyEyes lab vs. EasyEyes home).

The geometric mean (and SD log) was 3.06 (0.23) deg for EasyEyes lab, 3.00 (0.25) deg for EasyEyes home, and 3.5 (0.20) deg for CriticalSpacing.m lab (Figure 1B). Additionally, these estimates closely resembled the 50-observer crowding survey published by Kurzawski et al. (2023) 2.47 (0.16) deg.

### Individual differences are conserved across methods

**Figure 2:**
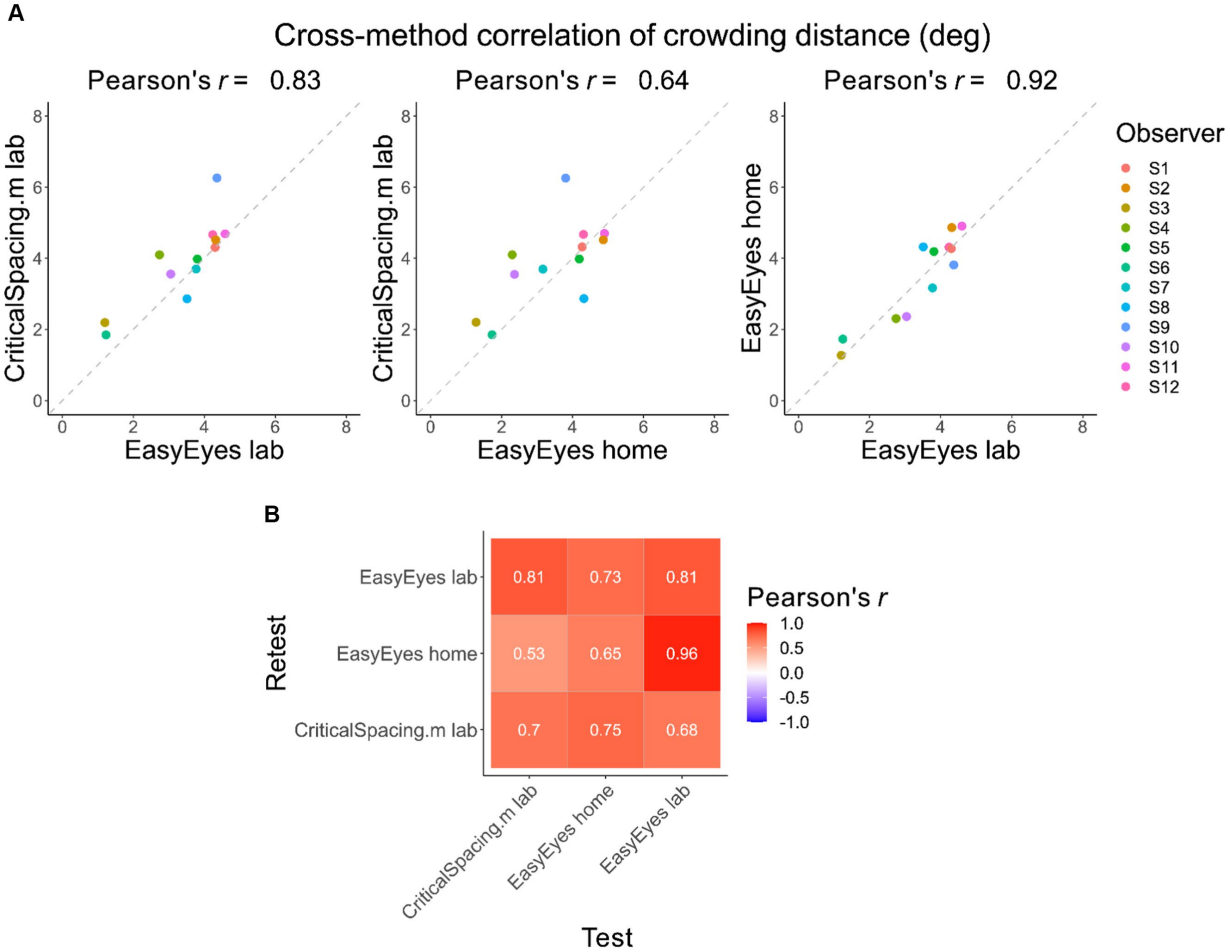
Correlations of crowding distance across methods. **(A)** The cross-method correlations of the geometric mean crowding distance for each observer. Mean is calculated from 4 thresholds (2 meridians, test–retest). **(B)** Test–retest Pearson’s correlation across mean crowding distance thresholds, across and within methods.

Here, we check whether individual differences are reproducible across the three methods. Pearson’s correlation coefficients across methods are high, showing that these differences were conserved (Figure 2A). Furthermore, the test–retest correlations within each method are not different from test–retest across methods (Figure 2B). This is indicated by similar values of Pearson’s rank correlation coefficients across the whole correlation matrix. To evaluate the consistency across all three methods, we calculated the intraclass correlation coefficient (ICC), which was 0.77 and indicates good reliability (Koo and Li, 2016).

### Fixational accuracy of EasyEyes

Observers are asked to use the cursor to track the moving crosshair, which they do quite well (RMSE of 0.08 deg). During tracking, the target appears at a random time (between 0.75 and 1.25 s), so the observer cannot predict when to look toward the anticipated target location. When the timed interval ends, the crosshair disappears, the cursor is hidden, and the target appears.

We used gaze tracking to monitor how well this foveal tracking task achieves correct fixation during stimulus presentation. For reference, we similarly analyze the conventional awaited-fixation method (gaze-contingent stimulus presentation), which uses gaze tracking (Kreyenmeier et al., 2020; Hanning and Deubel, 2022; Hanning et al., 2022; Kurzawski et al., 2023; Kwak et al., 2023).

**Figure 3:**
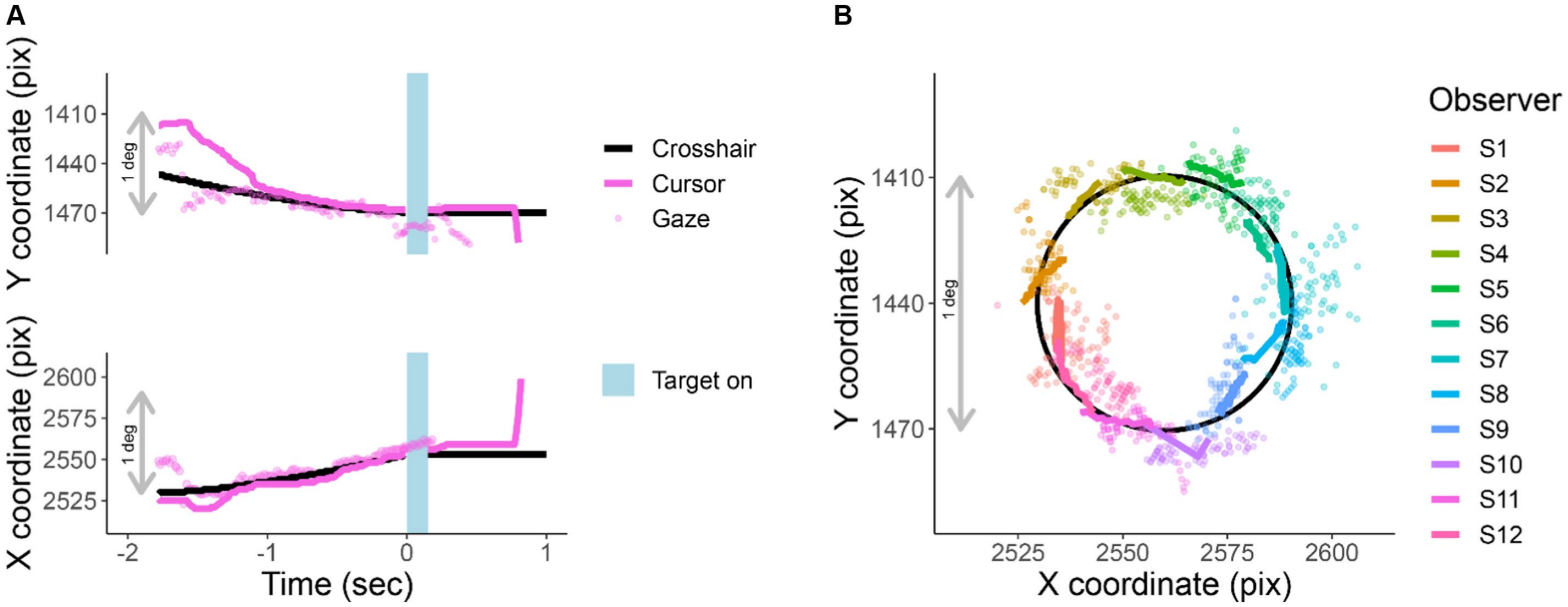
Cursor tracking task. X and Y positions of crosshair (solid black line), cursor (solid colored line), and gaze (colored points) during an EasyEyes trial. The gray bar corresponds to 1 deg (60 pix/deg). **(A)** X and Y coordinates as a function of time relative to the stimulus onset of one observer. The light blue bar represents the target duration (150 ms). **(B)** Shows a single representative trial from each observer (750 ms before target onset). The black circle is the trajectory of the crosshair. Again, thick colored lines indicate cursor, and colored dots indicate gaze position. Each observer’s data have been rotated around a circle (crosshair’s trajectory) to minimize overlap with other observers. The pink trial in **(A)** corresponds to S12 plotted in **(B)**. All X and Y positions have been corrected for estimated calibration bias.

While observers are tracking, their gaze remains near the crosshair (RMS of 0.6 deg). Figure 3A shows X and Y screen coordinates of gaze, cursor, and crosshair position as a function of time relative to stimulus onset. Figure 3B traces the X and Y position of gaze, crosshair, and cursor for one trial per participant during the last 750 ms of tracking.

### Reliability of fixation with EasyEyes

**Figure 4:**
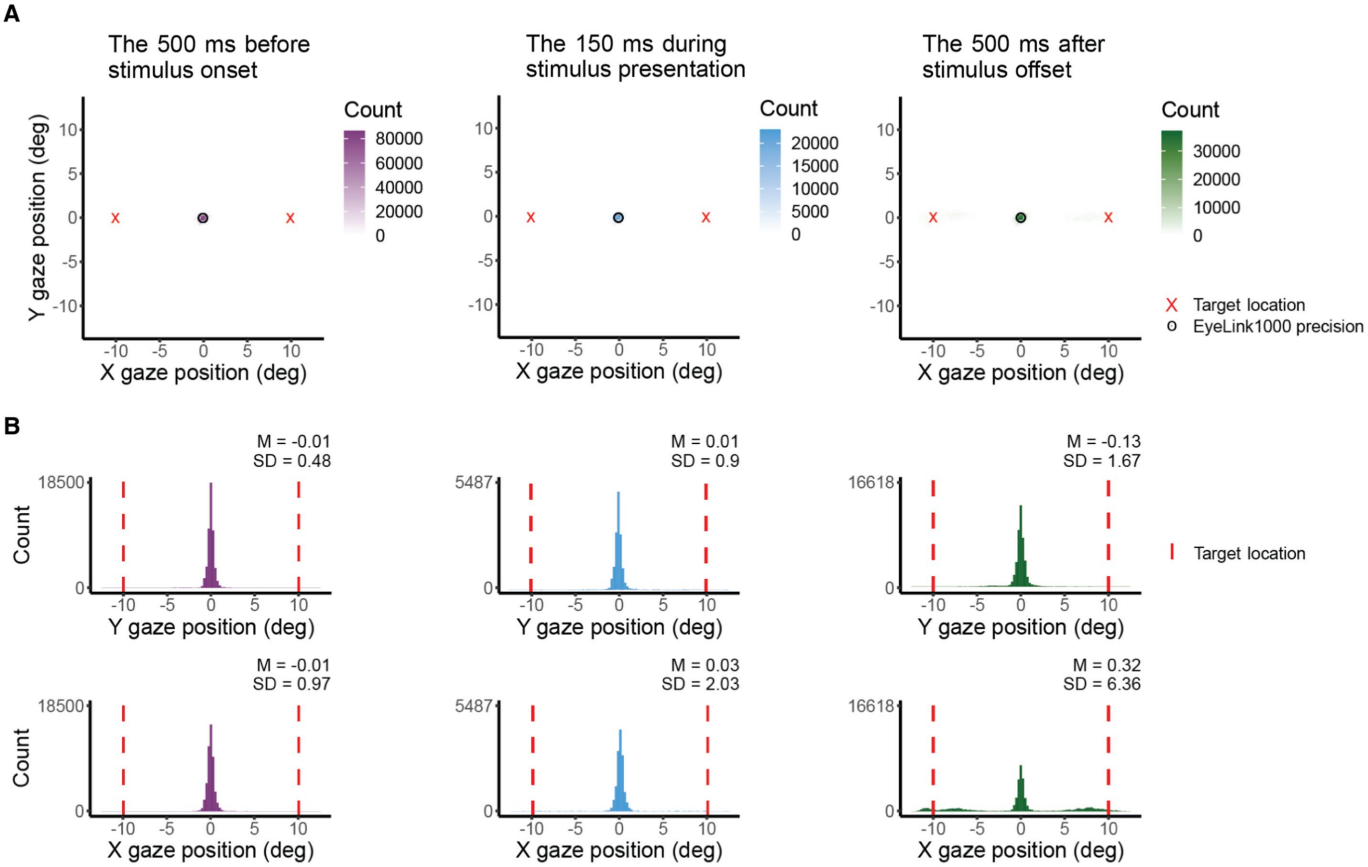
X and Y coordinates (in deg) of gaze before, during, and after target presentation across participants. **(A)** Shows 2D histograms of gaze. The circle indicates the eye tracker precision and the red cross is the target location. Total counts differ due to variations in the amount of data presented (500 ms vs. 150 ms) and the differences in the count of the bin with max counts. The 500 ms period is entirely within the 700 ms between target offset and response instructions. **(B)** Shows gaze position in X and Y coordinates. The red vertical line indicates the target location.

Figure 3B presents single-trial gaze tracking before stimulus presentation. Figure 4 shows gaze (visual coordinates), before, during, and after the stimulus presentation. Visual coordinates are in degrees relative to the center of the crosshair. The mean gaze position (obtained every 10 ms) across all trials (140), and all 12 observers is within 0.03 deg of the crosshair before and during the stimulus presentation, and within 0.3 deg after the stimulus presentation. The standard deviation is the lowest in the pre-stimulus interval, while observers are tracking (SD X = 0.97 deg, SD Y = 0.48 deg), increases during stimulus presentation (SD X = 2.03 deg, SD Y = 0.9 deg) and is highest after stimulus offset (SD X = 6.36 deg, SD Y = 1.67 deg). The higher standard deviation in horizontal direction before and during stimulus presentation reflects the overall tendency of eye movements to be directed horizontally (Engbert, 2006; Najemnik and Geisler, 2008; Otero-Millan et al., 2013; Rolfs and Schweitzer, 2022). The pronounced variance in horizontal gaze position after stimulus offset indicates (saccadic) eye movements toward the (now absent) target—a phenomenon commonly referred to as “looking at nothing” (Ferreira et al., 2008). Note that this gaze variance does not affect our perceptual measurement as the target was already undrawn. For gaze deviations during stimulus presentation, see Table 2.

**Table 2:** Standard deviations (in deg) of gaze position in X and Y coordinates during stimulus presentation for each participant using EasyEyes lab.

Observer	SD X (deg)	SD Y (deg)
S1	0.58	1.43
S2	0.45	0.37
S3	1.84	1.38
S4	1.02	0.93
S5	1.51	0.43
S6	0.31	0.16
S7	0.62	0.48
S8	0.45	0.52
S9	6.54	1.92
S10	0.53	0.34
S11	0.68	0.50
S12	0.37	0.25

**Figure 5:**
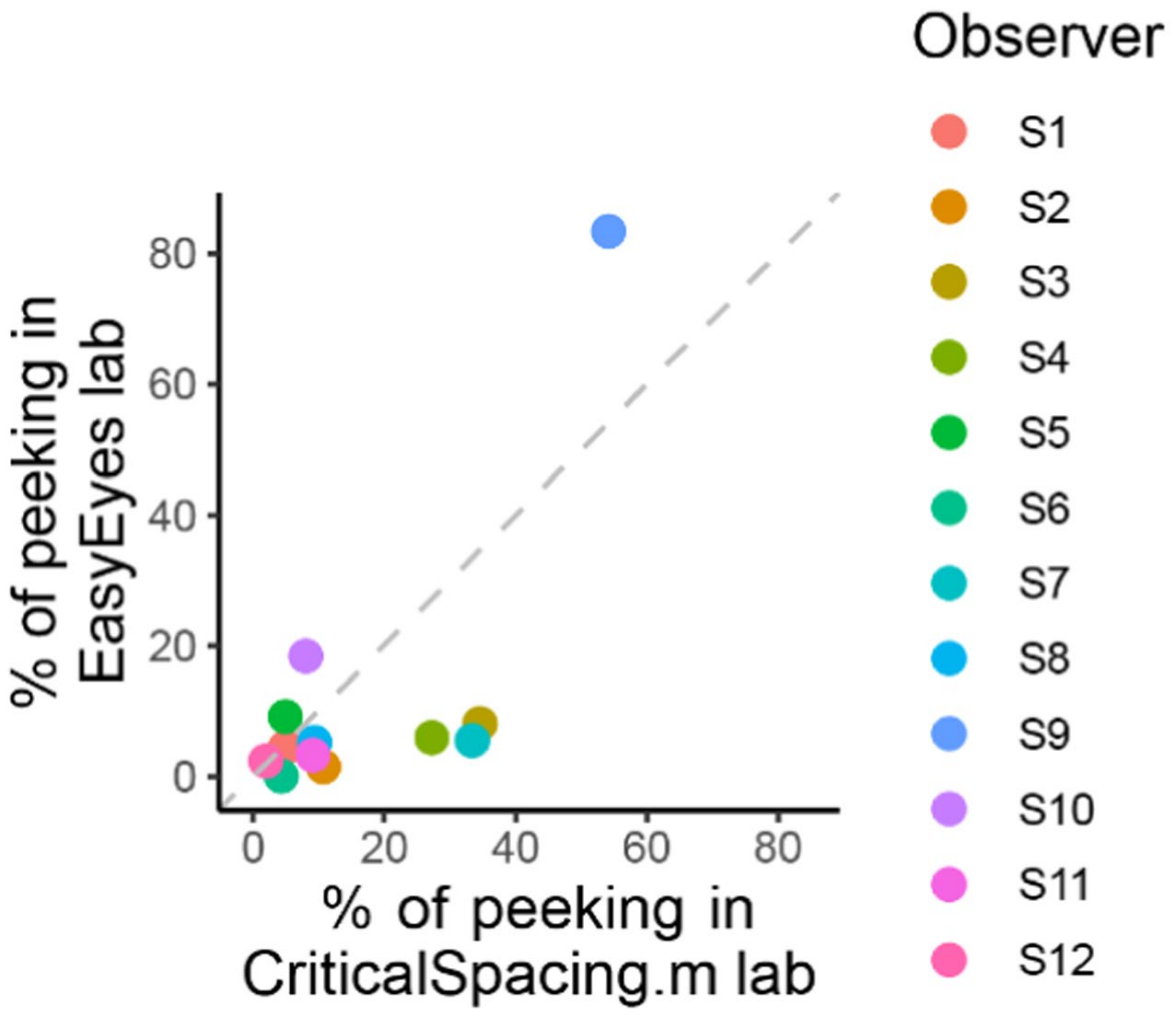
Comparing peeking across methods. The plot shows the percentage of trials in which observers peeked that is their gaze position was more than 1.5 deg away from the crosshair during stimulus presentation. For CriticalSpacing.m lab, peeks are detected by the eye tracker and correspond to rejected trials. For EasyEyes lab, we use gaze data to calculate the percentage of peeks post-hoc.

One out of 12 observers (S9) has a much higher standard deviation (Table 2) and often peeked at approximately 120 ms after stimulus onset. The short latency indicates that this participant has planned the eye movements with target onset. The same observer also peeked during many CriticalSpacing.m lab trials where they were detected and rejected by gaze tracking (Figure 5). The 11 remaining observers showed negligible peeking.

### Less peeking with moving crosshair task than with static fixation

CriticalSpacing.m lab counts the peeks and repeats the trials in which observers peeked. While using EasyEyes lab, we monitored gaze position to count each observer’s peeks. In the last section, we showed that observers fixate near the crosshair while tracking it with a cursor. Here, we wondered whether the tracking also reduces the urge to peek. We compared how often observers peek using a stationary crosshair method in CriticalSpacing.m lab versus the moving crosshair tracking task with EasyEyes lab. A peek is deemed to occur when the observer’s gaze deviates more than 1.5 deg from the last crosshair center position during stimulus presentation. The tracking task roughly halved the number of peeks across observers (median decreased from 9.3% to 5.4%). Individual data are shown in Figure 5. Even with a rather short stimulus duration (150 ms), one observer managed to peek with both methods. However, their peeking did not change their mean crowding distance relative to other observers. Furthermore, their thresholds were consistent across methods even though CriticalSpacing.m lab rejects peeking trials and EasyEyes cannot.

## Discussion

EasyEyes offers a new task to achieve accurate fixation online. We evaluated the accuracy of fixation and compared crowding thresholds measured online with EasyEyes or with our in-lab method (Kurzawski et al., 2023).

We tested 12 naive observers using traditional fixation and gaze-contingent stimulus display (CriticalSpacing.m lab), and using EasyEyes online at home (EasyEyes home) as well as in the laboratory while independently monitoring gaze (EasyEyes lab). Comparing the mean and standard deviation in thresholds across observers, we do not find significant differences across methods. Cross-method and within-method correlations are not different and individual differences are conserved. With a gaze tracker, we validate that EasyEyes achieves accurate fixation during target presentation.

### Importance of accurate fixation

Visual sensitivity decays with increasing distance from the fovea (the center of gaze). The density of photoreceptor and midget retinal ganglion cells declines with retinal eccentricity, increasing receptive field size (Freeman and Simoncelli, 2011; Anton-Erxleben and Carrasco, 2013). Thus, the visual system loses sensitivity (e.g., to higher spatial frequencies, contrast, or orientation changes) in the periphery. As a consequence, performance in nearly all visual tasks scales with the retinal eccentricity of the test stimulus [but see Hanning and Deubel (2022) for an eccentricity-independent approach]. Visual crowding is no exception: crowding distance scales linearly with eccentricity (Bouma, 1970). In order to achieve a stable threshold estimate, precise control of fixation is indispensable to ensure consistent measurement at the desired retinal eccentricity.

### Gaze during cursor tracking

Previous research has shown that successful tracking of a moving object with a hand-controlled cursor requires that gaze should closely follow the moving object (Xia and Barnes, 1999; Niehorster et al., 2015; Danion and Flanagan, 2018). Based on this, we asked observers to use the cursor to track the moving crosshair with the goal of keeping their gaze near the crosshair. Indeed, all of our observers use the cursor reliably to track the crosshair, keeping their gaze near both. This ensures the desired retinal target eccentricity. Both gaze and hand tend to lag the target (Koken and Erkelens, 1992).

### Classifying peekers

Researchers with an eye tracker can filter “peeking” behavior by making stimulus presentation contingent on fixation or using gaze position to remove trials where peeking occurred post-hoc. Our novel method of ensuring fixation gets around the need for eye tracking, so new methods are needed to filter out “peekers.” Within our sample (*N* = 12), one observer (S9) peeked. S9 had the highest RMSE between the crosshair and cursor and the most frames with unsuccessful tracking, suggesting that peeking and tracking behavior are associated. One may use an RMSE criterion to predict peeking from tracking behavior (we thank Reviewer 2 for this suggestion), but more data would be required to warrant this a valid approach for classifying participants as peekers and non-peekers. In our data, post-hoc omitting the 20% of observers with the highest crosshair-cursor RMSE effectively eliminates peekers. However, toward the goal of assessing crowding as a biomarker, one must consider both subpopulations and analyze them separately.

As peeking reduces crowding distance (Kurzawski et al., 2023), S9 had a lower crowding distance when peeking trials were not excluded (Compare EasyEyes home and laboratory with CriticalSpacing.m in Figure 2). This highlights the need to classify peekers and non-peekers.

### Comparing to previous work

Our study is very similar to Kurzawski et al. (2023)—both used CriticalSpacing.m without crosshair tracking, so differences in results cannot be attributed to differences in task load. There were several minor differences in methods: The 50 participants in Kurzawski et al. were psychology graduate students experienced in peripheral testing, while here we recruited 12 adults in the university area with no prior experience in psychophysical testing. Here, the geometric mean crowding threshold and the test–retest standard deviation are slightly higher than reported by Kurzawski et al. (geometric mean of 3.5 vs. 2.5 deg; test–retest standard deviation of 0.16 vs. 0.11). The more experienced observers had log thresholds with lower mean and standard deviation. A closer look reveals that the test–retest ratio is lower for naive participants (Table 2), indicating that they improved more from the first to the second threshold measurement. This is consistent with previous accounts of the effect of practice on lowering crowding distance (Chung, 2007). Despite these differences between the current study and Kurzawski et al., the consistency across results between methods is high, and individual differences in crowding distance are conserved.

### Why measure crowding (online)?

Both crowding distance and acuity are roughly proportional to eccentricity (Bouma, 1970) and thus are similarly sensitive to errors in fixation. We are not aware of any test that is more sensitive to eccentricity. Ophthalmology and optometry clinics routinely measure acuity. Here, we explore the possibility that they might find it worthwhile to also measure crowding. Foveal acuity determines the smallest text size that can be read at a certain eccentricity, and peripheral crowding puts an upper limit on reading speed (Pelli et al., 2007). Kurzawski et al. (2023) found hardly any correlation (*r* = 0.15) between foveal and peripheral crowding. Because of its sensitivity to eccentricity and its potential clinical utility, peripheral crowding is a suitable measurement to validate EasyEyes.

From a scientific point of view, accurate fixation for online vision testing enabled by EasyEyes will help to scale up our study of crowding as a promising biomarker of the development and health of the visual cortex. Crowding is correlated with dyslexia (Kwon et al., 2007) and can be measured years before the child learns to read (Pelli and Tillman, 2008). Besides this, online testing will facilitate cross-sectional and longitudinal surveys of crowding and related measures. Based on its correlation with dyslexia, we also anticipate a correlation between crowding and reading speed, and that pre-literate crowding might predict later reading speed.

### Quality of online data

There have been many evaluations of online testing data quality. Some of these reports find comparable data quality between online and in-lab studies (e.g., Goodman et al., 2013; Gureckis et al., 2016). Others identify serious problems with online data (e.g., McGonagle, 2015; Smith et al., 2016). To deal with this, it is often recommended to include tests to screen out observers who are not fully engaged. Fortunately, threshold tasks are good at screening out non-attentive participants, as they yield very high thresholds, unless an observer is attending reliably (Freeman et al., 2013; Majaj et al., 2015). Answering an easy trial incorrectly tends to produce a high threshold estimate that stands out as an outlier. Furthermore, the crosshair tracking task requires successful tracking for target presentation, which demands full attention.

### Why test online?

Online testing allows researchers to test hundreds or thousands of participants in a day, recruit diverse and special populations, and screen underserved populations. As online vision testing gains popularity, a new generation of testing software [e.g., jsPsych (de Leeuw, 2015), lab.js (Henninger et al., 2019), PsychoJS (Pitiot et al., 2017), Gorilla (Anwyl-Irvine et al., 2020), and OpenSesame (Mathôt et al., 2012)] makes it easier to test online than in the laboratory. However, established software lacks the possibility of using gaze tracking to achieve precise fixation—a requirement for most vision tests. Using the cursor to track a moving crosshair, EasyEyes delivers precise fixation—and the same thresholds online as we previously have measured in the lab. Our study shows that EasyEyes is a promising tool for lab-quality online vision testing. Despite other differences, such as the absence of supervision, diversity of equipment, and domestic distractions, EasyEyes achieves precise peripheral, fixation-dependent measurements that so far could only be obtained in the laboratory.

## Conclusion

Cursor tracking of a moving crosshair yields accurate fixation (RMSE of 0.6 deg). This method results in crowding thresholds equivalent to those measured in the lab with EyeLink 1000 gaze tracking. This trick facilitates online testing of any fixation-dependent measures. EasyEyes enables fixation-dependent measurements online, for easy testing of larger and more diverse populations.

## Data availability statement

The datasets presented in this study can be found in online repositories. The names of the repository/repositories and accession number(s) can be found at: https://osf.io/u6gdj/.

## Ethics statement

The studies involving humans were approved by Committee on Activities Involving Human Subjects. The studies were conducted in accordance with the local legislation and institutional requirements. The participants provided their written informed consent to participate in this study.

## Author contributions

JK: Conceptualization, Data curation, Formal analysis, Investigation, Methodology, Project administration, Resources, Software, Validation, Visualization, Writing – original draft, Writing – review & editing. MP: Conceptualization, Data curation, Formal analysis, Investigation, Methodology, Project administration, Resources, Validation, Visualization, Writing – original draft, Writing – review & editing. AB: Resources, Software, Writing – review & editing. NH: Software, Writing – review & editing, Resources. SL: Software, Writing – review & editing. NM: Conceptualization, Funding acquisition, Supervision, Writing – original draft, Writing – review & editing. DP: Conceptualization, Funding acquisition, Project administration, Resources, Software, Supervision, Writing – original draft, Writing – review & editing.

## Footnotes

^1^ https://easyeyes.app/

^2^ https://pavlovia.org/

^3^ https://www.prolific.co/
